# Cardiovascular outcomes and healthcare costs of liraglutide versus basal insulin for type 2 diabetes patients at high cardiovascular risk

**DOI:** 10.1038/s41598-020-80753-9

**Published:** 2021-01-14

**Authors:** Wan-Chun Huang, Yen-Chou Chen, Chung-Hsuen Wu, Yu Ko

**Affiliations:** 1 School of Pharmacy, College of Pharmacy, Taipei Medical University, No.250, Wuxing St., Taipei, 11031 Taiwan; 2 Division of Cardiovascular Medicine, Department of Internal Medicine, Wan Fang Hospital, Taipei Medical University, Taipei, Taiwan; 3 Research Center for Pharmacoeconomics, College of Pharmacy, Taipei Medical University, Taipei, Taiwan

**Keywords:** Endocrine system and metabolic diseases, Health care economics

## Abstract

We aimed to compare the (1) clinical outcomes including composite cardiovascular outcomes, cardiovascular death, and all-cause death, and (2) healthcare costs of using liraglutide and basal insulin as an initial treatment for patients with type 2 diabetes mellitus (T2DM) and high cardiovascular diseases (CVD) risk. This is a retrospective cohort study using Taiwan’s Health and Welfare Database. A total of 1057 patients treated with liraglutide were identified and matched with 4600 patients treated with basal insulin. The liraglutide group had a lower risk of a composite CVD outcome (hazard ratio (HR) 0.65; 95% confidence interval (CI) 0.50–0.85; p < 0.01), all-cause mortality (HR 0.40; 95% CI 0.28–0.59; p < 0.0001), and nonfatal stroke (HR 0.54; 95% CI 0.34–0.87; p = 0.01). Compared to the basal insulin group, the liraglutide group had lower median per-patient-per-month (PPPM) inpatient, emergency room (ER), and total medical costs, but higher median PPPM outpatient, total pharmacy, and total costs (all p < 0.0001). In conclusion, compared to basal insulin, liraglutide was found to be associated with reduced risk of a composite CVD outcome, nonfatal stroke, and all-cause mortality among high CVD risk patients with T2DM. In addition, liraglutide users had lower inpatient, ER, and total medical costs, but they had higher outpatient and total pharmacy costs.

## Introduction

Diabetes mellitus is a complex metabolic disorder that affects more than 400 million people in the world^1,2^, with over 90% of those patients diagnosed with type 2 diabetes mellitus (T2DM)^3^. In Taiwan, there were more than two million people affected by diabetes in 2018^4^, and the prevalence has also increased in recent decades^5–7^. T2DM is a major risk factor for developing cardiovascular diseases (CVDs)^8^. Among patients worldwide with T2DM, approximately 32.2% are affected by CVDs, and more than 50% of total deaths resulted from CVDs^9^.

T2DM is also a huge burden on the healthcare system. According to the report of the International Diabetes Federation, the direct costs of diabetes were estimated at United States dollar (USD) 760 billion in 2019, and the costs are expected to reach USD 825 billion in 2030 and USD 845 billion in 2045^10^. In Taiwan, T2DM has been the second most costly disease in outpatient settings in recent years^4,11,12^, with a cost of NT$ 20 billion in 2018^4^. Diabetes-related complications also significantly increase healthcare costs and utilization. It has been estimated that complications account for 53% of total medical costs of patients with diabetes, and that diabetes-related macrovascular diseases account for a relatively high proportion of those medical costs compared to other complications^13^.

According to the American Diabetes Association guidelines^14^ for patients with T2DM and high CVD risk, glucagon like peptide-1 receptor agonists (GLP-1 RAs) and sodium-glucose co-transporter 2 inhibitors that have cardiovascular benefits are suggested as second-line treatments. In Taiwan, all antidiabetic medicines with cardiovascular benefits are covered by the National Health Insurance (NHI) except semaglutide, and since liraglutide was the first one to be covered by the NHI, it provides a longer period for observing outcomes (liraglutide has been covered by the NHI since 2012 while the other drugs have been covered since 2016). As such, liraglutide was chosen to be investigated for its real-world cardiovascular benefits in this study.

In the Liraglutide Effect and Action in Diabetes: Evaluation of Cardiovascular Outcome Results (LEADER) trial, liraglutide was associated with lower risks of a composite CVD outcome, cardiovascular mortality, and all-cause mortality in patients with T2DM and high CVD risk^15^, whereas basal insulin showed a neutral effect on CVD^16,17^. In a study of the healthcare costs of liraglutide, Shah et al. demonstrated that liraglutide combined with standard treatment was cost-effective over a lifetime horizon for T2DM patients with high CVD risk compared to standard care as a result of a reduction of event-related costs and an increase in both quality-adjusted life-years and life-years^18^. However, the long-term cardiovascular effects and healthcare costs of liraglutide treatment remain unclear in the real-world setting. In this study, we therefore aimed to compare liraglutide and basal insulin on (1) clinical outcomes, including a composite cardiovascular outcome, cardiovascular death, and all-cause death, and (2) healthcare costs from the perspective of Taiwan’s NHI.

## Methods

### Data sources

Data were derived from the Health and Welfare Database (HWD) provided by the Health and Welfare Science Center, Ministry of Health and Welfare. The study database is a de-identified and encrypted insurance claim database that covers more than 99% of Taiwan’s citizens and over 92% of its total hospitals and clinics^19^. All of the diseases and procedures in this study were identified by the International Classification of Diseases, Ninth Revision, Clinical Modification (ICD-9-CM) codes for dates prior to 2016 and by the International Classification of Diseases, Tenth Revision, Clinical Modification (ICD-10-CM) codes and the International Classification of Diseases, Tenth Revision, Procedure Coding System (ICD-10-PCS) for dates after 31 Dec 2015^20,21^. In addition, the cause of death was coded by ICD-10 while the medications were defined by the drug codes used by the NHI^22^.

### Study design

This study is a retrospective observational cohort study that aimed to evaluate the risk of cardiovascular events and mortality as well as healthcare costs of the high CVD risk patients with T2DM who initially used liraglutide compared to those who initially used basal insulin (including neutral protamine Hagedorn, insulin glargine and insulin detemir). The enrollment period for both groups was from October 1, 2012 (i.e., the date that the NHI started to cover liraglutide) to December 31, 2016. The index date was defined as the first date when liraglutide or basal insulin was prescribed during the enrollment period. Enrolled patients’ clinical characteristics; such as comorbidities, adapted Diabetes Complications Severity Index (aDCSI) score, and prescribed antidiabetic drugs; were determined from data collected in the year before index date whereas demographic information such as birth year, gender, and residential area were determined based on the HWD’s Registry for Beneficiaries file in January or February 2012. In addition, a period of 3 years before index date was employed to collect data to assess patients’ CVD risk, and T2DM duration was calculated from the first date of T2DM diagnosis to the index date using medical records back to January 1, 2000.

This study was approved by the Taipei Medical University-Joint Institutional Review Board (TMU-JIRB) (Approval number: N201709052) and all methods were performed in accordance with its guidelines and regulations. As the study data were de-identified, informed consent was waived by TMU-JIRB.

### Inclusion and exclusion criteria

Patients enrolled needed to be diagnosed with T2DM in at least one inpatient claim or three outpatient claims during the 365 days before their index date. Moreover, they needed to meet the definition of high CVD risk: at least two diagnoses of atherosclerosis, one diagnosis of atherosclerosis plus one related procedure (i.e., cardiac diagnostic catheterization, percutaneous coronary intervention, and coronary artery bypass grafting), or a diagnosis of myocardial infarction (MI), stroke, heart failure (HF), or peripheral vascular disease (PVD) within 3 years before index date. In addition, the subjects needed to be at least 20 years old on the index date. Subjects were excluded if they had type 1 diabetes mellitus, gestational diabetes, or diabetic ketoacidosis at any point between October 1, 2009 (three years before the enrollment period) and December 31, 2017 (the last day data was available). In addition, subjects who used liraglutide or basal insulin in the year before the index date were excluded (i.e., the wash-out period). Moreover, the subjects were also excluded if their birth year or residential area were unavailable.

### Outcome measures

The primary outcome for the assessment of effectiveness was the first event of a composite CVD outcome, consisting of MI, stroke, HF, and cardiovascular mortality (i.e., death from heart disease (ICD-10: I01–I02.0, I05–I09, I20–I25, I27, I30–I52) or cerebrovascular disease (ICD-10: I60–I69)). Each of these events individually along with all-cause mortality were the secondary outcomes. The follow-up period started at the index date and ended with the first occurrence of the study endpoints, which included a nonfatal cardiovascular event (i.e., MI, stroke, or HF), a fatal event (cardiovascular mortality, or all-cause mortality), or the last day of the study period (December 31, 2017). In the sensitivity analysis, we used the discontinuation of the treatment of interest as the study endpoint, i.e., the last date of a patient’s receiving liraglutide or basal insulin plus 30 days.

To evaluate healthcare costs between the liraglutide and basal insulin groups from the NHI’s perspective, the two groups were compared on their outpatient costs, inpatient costs, emergency room (ER) costs, total pharmacy costs, total medical costs, and total costs. Total pharmacy costs were defined as all medication-related expenses reimbursed by the NHI plus patients’ copayment for drugs dispensed in clinics, hospitals, and pharmacies. Total medical costs were total costs minus total pharmacy costs. The follow-up period for evaluating healthcare costs started with the index date and ended at the time of a fatal event or on the last day of the study period (December 31, 2017), whichever occurred first.

### Statistical analysis

Baseline characteristics were summarized using means and standard deviations for continuous variables and proportions for categorical variables. To examine baseline characteristics between the two groups before and after propensity score matching (PSM), t-test was used for continuous variables and chi-square test and Fisher’s exact test were used for categorical variables.

To balance the two groups’ patient characteristics at baseline and to decrease selection bias, we conducted PSM with nearest neighbor matching without caliper. The variables used to estimate the propensity score included age, gender, residential area, CVD-related diagnosis (i.e., atherosclerosis, MI, HF, stroke, PVD, hypertension, and hyperlipidemia), aDCSI score, combined comorbidity score, T2DM duration, and the type and number of prescribed antidiabetic medicines. The aDCSI score was used to measure severity of diabetes^23,24^. It consists of a score of 0, 1, or 2 for six diabetes complications (retinopathy, nephropathy, cerebrovascular, cardiovascular, PVD, and metabolic) and 0, or 1 for neuropathy, giving a total score that ranges from 0 to 13. The combined comorbidity score incorporated Charlson’s and Elixhauser’s comorbidity index scores^25^ and included a total of 32 conditions. Moreover, exact matching was employed for gender, residential area, and each high CVD risk disease.

After PSM, effectiveness was examined using the Kaplan–Meier curve and Cox proportional hazard model. Using a Kaplan–Meier curve with a log-rank test, we obtained the survival curves of primary and secondary outcomes from index date to the end of the follow-up period. To compare the effects on CVD and mortality between liraglutide and basal insulin, the Cox proportional hazard model based on time to event was performed to estimate the crude and adjusted hazard ratios (adjusted for age and the use of statins, anticoagulant agents, and antiplatelet agents after the index date).

Healthcare costs were estimated per-patient-per-month (PPPM) from the index date to the end of follow-up (death or the last day of the study period). To analyze the trend of healthcare costs, we also compared the two groups’ PPPM healthcare costs in each individual year of the follow-up period. As healthcare costs are usually right-skewed, the Wilcoxon rank sum test and generalized linear models with a gamma distribution and a log link were used for cost comparison. The generalized linear models were adjusted for all potential confounders used in PSM. In the HWD, the cost data are reported as points, and one point is usually close to NT$1 but varies by healthcare sectors. For ease of calculation, we assumed one point to be equal to NT$1.

Statistical significance was defined as a two-sided p-value of < 0.05. All analyses were performed using SAS software version 9.4 (SAS Institute, Cary, North Carolina, USA).

## Results

### Study sample

**Figure 1:**
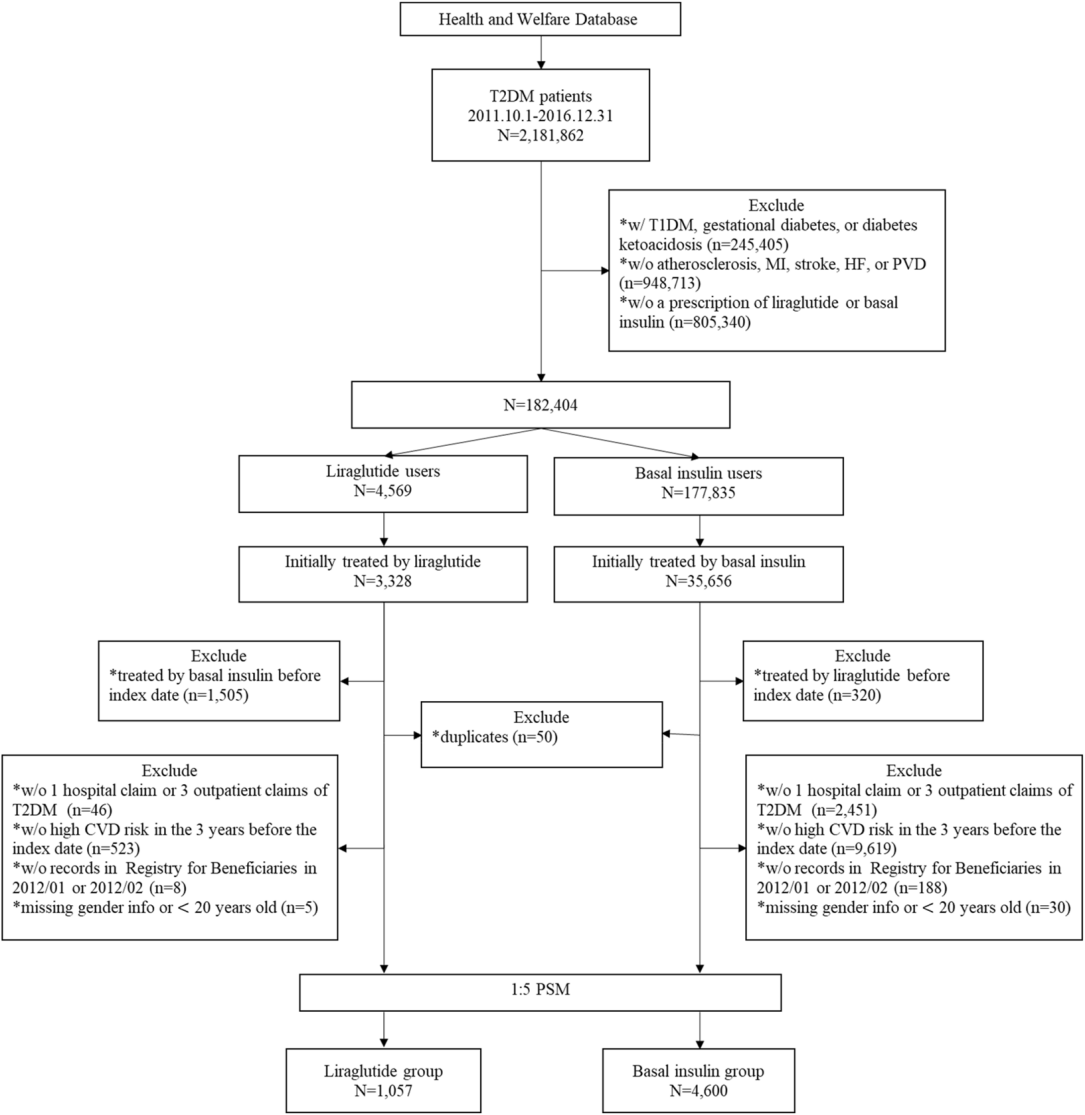
Flowchart of patient selection. *T2DM* type 2 diabetes mellitus, *T1DM* type 1 diabetes mellitus, *MI* myocardial infarction, *HF* heart failure, *PVD* peripheral vascular disease, *CVD* cardiovascular disease, *PSM* propensity score matching.

The process of patient selection is presented in Fig. 1. After PSM, there were 1,057 patients in the liraglutide group and 4600 patients in the basal insulin group. Table 1 summarizes the study patients’ characteristics before and after PSM. After PSM, no difference was found in any variables between the liraglutide group and the basal insulin group except age, prior use of GLP-1 RA, and history of MI. After PSM, the mean age was 57.0 years (standard deviation (SD): 11.3 years) and 58.3 years (SD: 11.0 years) in the liraglutide and basal insulin groups, respectively. In both groups, most of the patients lived in northern Taiwan (liraglutide: 58.2%; basal insulin: 58.8%), and about half were male. The mean combined comorbidity score was 0.6 and 0.5 points in the liraglutide and basal insulin groups, respectively, while the mean aDCSI score for both groups was 1.8 points and the mean duration of T2DM for both groups was 4.5 years. The most common prior CVDs were atherosclerosis, PVD, and HF. The most common antidiabetic medicines that had been used previously were biguanides, sulfonylureas, and dipeptidyl peptidase-4 inhibitors. In addition, slightly less than half of the study subjects in both groups had received more than four types of antidiabetic medicines.

**Table 1:** Study subjects’ baseline characteristics.

	Before PSM					After PSM				
	Basal insulin N = 22,998		Liraglutide N = 1191		p value	Basal insulin N = 4600		Liraglutide N = 1057		p value
	Mean	SD	Mean	SD		Mean	SD	Mean	SD	
Age (years)	66.2	12.3	56.2	11.6	< 0.0001	58.3	11.0	57.0	11.3	< 0.001
T2DM duration (years)	5.3	4.8	4.5	4.6	< 0.0001	4.5	4.8	4.5	4.6	0.88
Combined comorbidity score	1.4	2.7	0.6	2.3	< 0.0001	0.5	2.1	0.6	2.3	0.49
aDCSI score	2.1	1.6	1.8	1.4	< 0.0001	1.8	1.4	1.8	1.3	0.70

	N	%	N	%	p value	N	%	N	%	p value
**Gender**					0.11					0.78
Male	12,324	53.6	610	51.2		2381	51.8	542	51.3	
Female	10,674	46.4	581	48.8		2219	48.2	515	48.7	
**Residential area**				–	< 0.0001					0.43
Northern	9862	42.9	666	55.9		2706	58.8	615	58.2	
Central	6164	26.8	239	20.1		879	19.1	196	18.5	
Southern	6055	26.3	229	19.2		877	19.1	204	19.3	
Others	917	4.0	57	4.8		138	3.0	42	4.0	
**Comorbidities**										
Hypertension	16,136	70.2	863	72.5	0.09	3432	74.6	781	73.9	0.63
Hyperlipidemia	11,600	50.4	727	61.0	< 0.0001	2821	61.3	646	61.1	0.90
**Prior CVD history**										
Atherosclerosis	12,336	53.6	689	57.9	< 0.01	2724	59.2	616	58.3	0.58
PVD	8344	36.3	409	34.3	0.17	1500	32.6	358	33.9	0.43
HF	5609	24.4	251	21.1	0.01	806	17.5	205	19.4	0.15
Stroke	5088	22.1	129	10.8	< 0.0001	435	9.5	104	9.8	0.70
MI	1582	6.9	72	6.0	0.27	178	3.9	55	5.2	0.05
**Prior use of antidiabetic medicines**										
Biguanide	18,331	79.7	1082	90.8	< 0.0001	4151	90.2	957	90.5	0.77
SU	19,091	83.0	929	78.0	< 0.0001	3799	82.6	850	80.4	0.10
DPP4i	16,407	71.3	885	74.3	0.03	3551	77.2	817	77.3	0.95
α-glucosidase inhibitor	7853	34.1	349	29.3	< 0.001	1472	32.0	322	30.5	0.33
TZD	5960	25.9	333	28.0	0.12	1316	28.6	295	27.9	0.65
Insulin^b^	8009	34.8	313	26.3	< 0.0001	1138	24.7	282	26.7	0.19
Meglitinide	4756	20.7	162	13.6	< 0.0001	576	12.5	138	13.1	0.64
SGLT2i	86	0.4	16	1.3	< 0.0001	33	0.7	13	1.2	0.09
GLP-1RA^c^	25	0.1	74	6.2	< 0.0001	3	0.1	5	0.5	< 0.01^a^
**No. of antidiabetic medicines used**					< 0.0001					0.85
0	63	0.3	0	0		0	0	0	0	
	1055	4.6	28	2.4		92	2.0	25	2.4	
1 2	2997	13.0	147	12.3		542	11.8	126	11.9	
3	7627	33.2	461	38.7		1788	38.9	416	39.4	
≥ 4	11,256	48.9	555	46.6		2178	47.3	490	46.4	

^a^Fisher’s exact test.

^b^Excluding basal insulin.

^c^Excluding liraglutide; Standard deviation (SD); Type 2 Diabetes mellitus (T2DM); Adapted diabetes complications severity index (aDCSI); Cardiovascular disease (CVD); Peripheral vascular disease (PVD); Heart failure (HF); Myocardial infarction (MI); Sulfonylurea (SU); Dipeptidyl peptidase-4 inhibitor (DPP4i); Thiazolidinedione (TZD); Sodium-glucose co-transporter 2 inhibitor (SGLT2i); Glucagon like peptide-1 receptor agonist (GLP-1RA).

### Effectiveness

**Figure 2:**
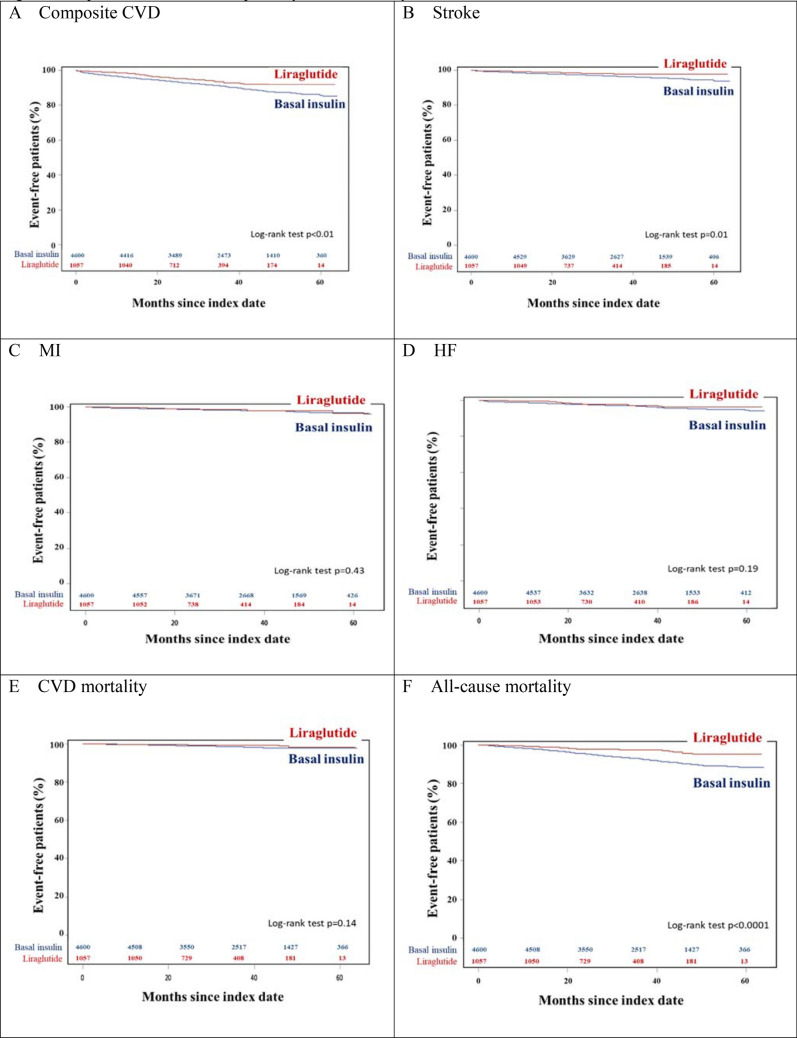
Kaplan–Meier curves of primary and secondary outcomes. Figure presents the survival curves of the primary and secondary outcomes from the index date to the end of the follow-up period. The outcomes are CVD composite (**A**), stroke (**B**), MI (**C**), HF (**D**), CVD mortality (**E**), and all-cause mortality (**F**).

As shown in Fig. 2 and Table 2, compared to the basal insulin group, patients in the liraglutide group had a lower likelihood of having a composite of CVDs (hazard ratio (HR) 0.65; 95% confidence interval (CI) 0.50 to 0.85; p < 0.01), stroke (HR 0.54; 95% CI 0.34 to 0.87; p = 0.01), and all-cause mortality (HR 0.40; 95% CI 0.28 to 0.59; p < 0.0001) while no difference was found in MI, HF, and CVD mortality. Similar results were observed after adjusting for age and use of statins, anticoagulant agents, and antiplatelet agents (composite of CVDs: adjusted hazard ratio (aHR) 0.67; 95% CI 0.52 to 0.88; p < 0.01; stroke: aHR 0.57; 95% CI 0.35 to 0.91; p = 0.02; all-cause mortality: aHR 0.45; 95% CI 0.31 to 0.65; p < 0.0001).

**Table 2:** Hazard ratios for primary and secondary outcomes.

	Basal insulin N = 4600		Liraglutide N = 1057		HR	p-value	95% CI	aHR	p-value	95% CI
	n	%	n	%						
**Primary outcome**										
Composite CVD	463	10.1	61	5.8	0.65	< 0.01	0.50–0.85	0.67	< 0.01	0.52–0.88
**Secondary outcomes**										
Stroke	177	3.8	19	1.8	0.54	0.01	0.34–0.87	0.57	0.02	0.35–0.91
MI	106	2.3	17	1.6	0.82	0.43	0.49–1.36	0.83	0.47	0.50–1.39
HF	173	3.8	26	2.5	0.76	0.19	0.50–1.15	0.80	0.27	0.52–1.20
CVD mortality	75	1.6	9	0.9	0.60	0.14	0.30–1.20	0.65	0.23	0.33–1.30
All-cause mortality	361	7.8	29	2.7	0.40	< 0.0001	0.28–0.59	0.45	< 0.0001	0.31–0.65

*HR* hazard ratio, *aHR* adjusted hazard ratio, *CI* confidence interval, *CVD* cardiovascular disease, *MI* myocardial infarction, *HF* heart failure.

In the sensitivity analysis, after adding the discontinuation of treatment as one of the study endpoints, the outcomes remained consistent with the primary analysis results (composite of CVDs: aHR 0.69; 95% CI 0.53 to 0.91; p = 0.01; stroke: aHR 0.56; 95% CI 0.35 to 0.90; p = 0.02; all-cause mortality: aHR 0.45; 95% CI 0.31 to 0.66; p < 0.0001).

### Estimation of healthcare costs

The results of Wilcoxon rank sum tests are shown in Table 3. The median PPPM inpatient costs (NT$ 0 vs. NT$ 321.9; p < 0.0001), ER costs (NT$ 32.9 vs. NT$ 58.4; p < 0.0001), and total medical costs (NT$ 2338.1 vs. NT$ 2792.3; p < 0.0001) were lower in patients treated with liraglutide than those on basal insulin. In contrast, the median PPPM outpatient costs (NT$ 7199.0 vs. NT$ 5285.5; p < 0.0001), total pharmacy costs (NT$ 5,481.4 vs. NT$ 3,414.6; p < 0.0001), and total costs (NT$ 8,208.8 vs. NT$ 6506.3; p < 0.0001) were higher in patients in the liraglutide group than those in the basal insulin group. The results of the analyses of healthcare costs in each individual year during the follow-up period were similar to those from the analysis of the whole follow-up period except that the differences between the two groups in outpatient costs and total costs gradually decreased over time until no significant difference was found in the 5th year (Online Appendix 1).

**Table 3:** PPPM costs (liraglutide vs. basal insulin).

	Basal insulin N = 4600			Liraglutide N = 1057			
	Median	Mean	SD	Median	Mean	SD	p value
Outpatient costs	5285.5	7685.0	9266.8	7199.0	8658.8	7626.1	< 0.0001
Inpatient costs	321.9	4310.1	16,578.4	0	2614.2	10,386.1	< 0.0001
ER costs	58.4	346.8	1010.8	32.9	248.8	985.3	< 0.0001
Total pharmacy costs	3414.6	4458.0	5484.4	5481.4	5905.1	3645.5	< 0.0001
Total medical costs	2792.3	7883.8	17,664.9	2338.1	5616.7	12,319.3	< 0.0001
Total costs	6506.3	12,341.9	20,697.9	8208.8	11,521.8	14,087.7	< 0.0001

*SD* standard deviation, *ER* emergency room.

Total costs = total pharmacy costs + total medical costs.

The generalized linear model was used to compare the two groups’ outpatient costs, total pharmacy costs, total medical costs, and total costs, adjusting for all potential confounders used in PSM. The results showed that patients treated with liraglutide had lower PPPM total medical costs but higher outpatient and total pharmacy costs than basal insulin users. The PPPM total costs were similar between the two groups.

## Discussion

In this study, we aimed to compare the risks of CVD and mortality as well as healthcare costs between liraglutide and basal insulin in patients with T2DM and high CVD risk. This study has several strengths. First, this retrospective cohort study is the first one to investigate the long-term cardiovascular effects and healthcare costs of liraglutide on a national level. Second, our data source is the HWD, which contains the claims data of more than 99% of Taiwan’s citizens. Hence, our study subjects are representative of the population in Taiwan. Third, this study confirmed the clinical outcomes observed in the LEADER trial in a real-world setting. Lastly, our study investigated the CVD effect of liraglutide in patients with a broader age range (22 to 97 years old) while the LEADER trial only assessed patients over 50 years old.

The effectiveness results in our study were similar to those in the LEADER trial^15^ except for CVD mortality and stroke. Both the LEADER trial and our study showed that patients treated with liraglutide had lower risks of a composite CVD outcome and all-cause mortality. However, our liraglutide group did not have a lower likelihood of CVD mortality as observed in the LEADER trial. An explanation for this could be the different definitions of death from CVD causes. In the trial, besides death from sudden cardiac death, MI, HF, and stroke, the trial also included the patients who died from other cardiovascular causes and the ones without a clearly documented non-vascular cause. In our study, however, only the subjects who were coded with heart disease or cerebrovascular disease in the Cause of Death Data were counted as CVD deaths. This stringent definition might have caused liraglutide’s lower CVD mortality to fail to reach statistical significance.

The other difference is the risk of stroke. In our study, the risk of stroke in the liraglutide group was significantly lower, whereas the LEADER trial reported a lower risk that was not significant (HR 0.89; 95% CI 0.72 to 1.11; p = 0.30). The different characteristics of the study patients probably contributed this difference. In the LEADER trial, patients enrolled in the study were over 50 years old with prior CVD history or over 60 years old with a CVD risk factor whereas our study only included patients with a CVD history. In the post-hoc analysis of the LEADER trial^26^, Verma et al. reported that the patients with CVD risk factors alone had a lower incidence rate of all CVD outcomes than those with a prior stroke or MI history. Moreover, in the subgroup of patients with only CVD risk factors, the effects of liraglutide on reducing risk were not significant. The failure to observe an effect of liraglutide in patients with T2DM and cardiovascular risk factors alone may have partially resulted from the lower incidence rate of CVD outcomes in this subgroup. Our findings were similar to the retrospective study by Zimmerman et al.^27^, which enrolled 105,862 T2DM patients to compare GLP-1 RA exposure to no exposure in a real-world setting. They found that GLP-1 RA exposure had a lower risk for a composite outcome (MI, stroke, and all-cause mortality), stroke, and all-cause mortality, but not for MI, just like our study.

The findings of our healthcare cost evaluation were comparable to those of previous studies that investigated other GLP-1 RAs. For example, a real-world study in the U.S. conducted by Mody et al. reported that dulaglutide users had lower medical costs and higher pharmacy costs during the 1-year follow-up period, compared to basal insulin users^28^. Another study by Wittbrodt et al. in the U.S. compared the healthcare costs of exenatide once weekly with insulin glargine and revealed that patients on exenatide had significantly lower medical costs and higher pharmacy costs^29^. As such, the high drug cost of GLP-1 RAs seems to be offset by their better clinical outcomes and consequently lower overall medical costs.

This study had several limitations. First, due to a lack of laboratory data in the HWD, we were not able to adjust for factors such as levels of systolic blood pressure, total cholesterol, and HDL cholesterol that were considered to be important predictors of subsequent coronary heart disease. Instead, we used the records of diagnoses (e.g., hypertension and hyperlipidemia) and medications prescribed (e.g., statins) as substitutes to control for these risk factors. In addition, although BMI also has an association with MI and stroke, we were unable to control for this CVD risk factor because the data were not available. Second, we used ICD-9-CM, ICD-10-CM, and ICD-10-PCS codes to define the diseases and procedures because the coding system was revised to the 10th version in 2016. There might be inconsistencies between the two coding systems, although we converted the codes mainly based on the NHI’s conversion tables and the methods used in previous studies, also taking into account expert opinions. Third, coding errors of diagnoses, procedures, and medications as well as missing data may exist in the study claim database. Fourth, patient adherence information was unavailable in the HWD and so could not be assessed. Lastly, this study examined the cardiovascular effect of liraglutide only among T2DM patients with a high CVD risk. The findings should not be extrapolated to all T2DM patients.

## Conclusion

In this retrospective cohort study, compared to basal insulin, T2DM patients with high CVD risk who were treated with liraglutide demonstrated a lower risk of a composite CVD outcome, stroke, and all-cause mortality. Moreover, liraglutide users had lower inpatient, ER, and total medical costs but higher outpatient, and total pharmacy costs than basal insulin users.

## Supplementary Information

Supplementary Information.

## Data Availability

The data used in the study from Health and Welfare Database was not publically available.
