# Supplementary material for: Cardiovascular outcomes and healthcare costs of liraglutide versus basal insulin for type 2 diabetes patients at high cardiovascular risk
Source: Sci Rep. 2021 Jan 14;11:1430. doi: 10.1038/s41598-020-80753-9 (PMC7809152; doi:10.1038/s41598-020-80753-9)
Supplement: Supplementary file 1 — Supplementary Information. [file 41598_2020_80753_MOESM1_ESM.docx]

Cardiovascular outcomes and healthcare costs of liraglutide versus basal insulin for type 2 diabetes patients at high cardiovascular risk

Wan-Chun Huang^1^, Yen-Chou Chen^2^, Chung-Hsuen Wu^1^, Yu Ko^1,3^

^1^School of Pharmacy, College of Pharmacy, Taipei Medical University, Taipei, Taiwan

^2^Division of Cardiovascular Medicine, Department of Internal Medicine, Wan Fang Hospital, Taipei Medical University, Taiwan

^3^Research Center for Pharmacoeconomics, College of Pharmacy, Taipei Medical University

Appendix 1. Time trend of various types of PPPM costs (liraglutide vs. basal insulin)

|  | 1^st^ Year | | 2^nd^ Year | | 3^rd^ Year | | 4^th^ Year | | 5^th^ Year | |
| --- | --- | --- | --- | --- | --- | --- | --- | --- | --- | --- |
|  | Basal insulin  N=4,600 | Liraglutide  N=1,057 | Basal insulin  N=4,508 | Liraglutide  N=1,050 | Basal insulin  N=3,542 | Liraglutide  N=729 | Basal insulin  N=2,515 | Liraglutide  N=407 | Basal insulin  N=1,427 | Liraglutide  N=180 |
| Outpatient costs | | | | | | | | | | |
| Median | 5,435.0* | 8,242.2* | 4,943.0* | 6,513.2* | 4,898.3* | 5,949.8* | 4,621.1* | 5,407.3* | 3,971.5 | 4,060.6 |
| Mean | 7,584.5 | 9,675.2 | 7,206.4 | 7,570.5 | 7,347.5 | 6,877.9 | 7,036.9 | 6,717.3 | 6,231.5 | 5,135.1 |
| SD | 9,296.1 | 8,300.0 | 9,651.7 | 6,864.6 | 10,142.5 | 6,747.9 | 10,299.8 | 8,020.8 | 9,554.3 | 6,607.5 |
| Inpatient costs | | | | | | | | | | |
| Median | 0* | 0* | 0 | 0 | 0* | 0* | 0* | 0* | 0 | 0 |
| Mean | 4,075.4 | 1,843.5 | 3,495.1 | 3,139.5 | 3,315.1 | 2,268.9 | 3,705.9 | 1,450.1 | 2,258.0 | 1,729.5 |
| SD | 16,789.0 | 7,416.4 | 28,198.8 | 18,828.2 | 16,311.4 | 10,883.1 | 19,952.8 | 5,921.6 | 10,305.1 | 6,604.5 |
| ER costs | | | | | | | | | | |
| Median | 0* | 0* | 0 | 0 | 0 | 0 | 0 | 0 | 0* | 0* |
| Mean | 325.6 | 220.1 | 273.6 | 213.4 | 289.1 | 234.9 | 271.6 | 182.0 | 222.3 | 67.5 |
| SD | 1,180.3 | 1,002.3 | 982.6 | 717.7 | 1,014.4 | 798.2 | 918.1 | 528.6 | 898.6 | 362.9 |
| Total pharmacy costs | | | | | | | | | | |
| Median | 3,573.4* | 6,354.7* | 3,182.2* | 4,885.1* | 3,122.7* | 4,315.3* | 2,954.1* | 3,531.7* | 2,407.8* | 2,742.2* |
| Mean | 4,581.7 | 6,698.5 | 4,058.7 | 5,426.6 | 4,051.0 | 4,648.8 | 3,783.4 | 4,110.7 | 3,012.9 | 3,265.9 |
| SD | 5,626.8 | 3,538.2 | 5,557.7 | 5,095.7 | 5,930.3 | 3,865.6 | 5,532.5 | 4,215.9 | 3,633.7 | 2,862.6 |
| Total medical costs | | | | | | | | | | |
| Median | 2,234.6* | 2,053.8* | 1,946.2 | 1,808.6 | 1,860.5* | 1,614.7* | 1,670.2 | 1,588.0 | 1,293.1* | 949.5* |
| Mean | 7,403.9 | 5,040.4 | 6,916.4 | 5,496.8 | 6,900.7 | 4,732.7 | 7,230.9 | 4,238.7 | 5,698.8 | 3,666.2 |
| SD | 17,686.0 | 11,099.2 | 28,610.9 | 16,896.0 | 17,386.5 | 11,874.4 | 19,586.4 | 9,476.9 | 14,056.4 | 9,171.1 |
| Total costs | | | | | | | | | | |
| Median | 6,186.4* | 8,843.0* | 5,489.2* | 7,137.0* | 5,440.4* | 6,454.2* | 5,120.2 | 5,912.4 | 4,281.8 | 4,552.2 |
| Mean | 11,985.6 | 11,738.8 | 10,975.0 | 10,923.4 | 10,951.7 | 9,381.6 | 11,014.3 | 8,349.4 | 8,711.8 | 6,932.1 |
| SD | 20,726.7 | 12,226.5 | 30,624.4 | 20,345.6 | 20,536.9 | 13,637.3 | 23,181.9 | 11,181.2 | 15,367.1 | 10,184.3 |

*p<0.05; Standard deviation (SD); Emergency room (ER)
